# Short Supply-Driven Modifications to Blood Culture Practice and Their Clinical Impact: A Single-Center Time-Series Study

**DOI:** 10.7759/cureus.88508

**Published:** 2025-07-22

**Authors:** Daisuke Ono, Kazuyuki Mimura, Yuki Watanabe, Eiyu Ebata, Kei Yamamoto, Yusuke Nishida, Yasufumi Suzuki, Masumi Ogawa, Shinichiro Fukuda, Kunihisa Tsukada, Hideaki Oka, Kyosuke Takeshita

**Affiliations:** 1 Infectious Diseases and Infection Control/Infectious Diseases, Saitama Medical Center, Saitama Medical University, Saitama, JPN; 2 General Internal Medicine, Saitama Medical Center, Saitama Medical University, Saitama, JPN; 3 Clinical Laboratory, Saitama Medical Center, Saitama Medical University, Saitama, JPN; 4 Clinical Laboratory/Microbiology, Saitama Medical Center, Saitama Medical University, Saitama, JPN; 5 General Internal Medicine/Infectious Diseases, Saitama Medical Center, Saitama Medical University, Saitama, JPN; 6 Medical Informatics, Saitama Medical Center, Saitama Medical University, Saitama, JPN; 7 Internal Medicine, Self-Defense Forces Central Hospital, Tokyo, JPN; 8 Pharmacy, Saitama Medical Center, Saitama Medical University, Saitama, JPN

**Keywords:** antimicrobial stewardship, blood culture bottle, bloodstream infections, data-driven protocols, supply shortage

## Abstract

Introduction: Blood cultures are essential for identifying bloodstream infections and guiding treatment. A global BD BACTEC™ bottle (BD, Franklin Lakes, NJ, US) shortage disrupted diagnostics, including at the Saitama Medical Center. During the shortage period, clinicians were instructed to prioritize cultures based on clinical necessity and infection focus, often opting for single-set submissions or deferring testing. This study examined changes in blood culture practices during the shortage period.

Methods: We conducted a retrospective observational study to analyze the blood culture data collected between January 1 and October 31, 2024. Five outcome indicators were evaluated: submission count, single-set submission rate, submission episode count, true-positivity rate, and contamination rate. The analyses were limited to patients aged ≥15 years to minimize pediatric-related biases. Segmented regression with Prais-Winsten adjustment was used to perform interrupted time-series analysis and account for autocorrelation and seasonal trends. Additionally, the detection frequencies of true bloodstream pathogens were compared between the pre-shortage and shortage periods using Fisher’s exact test.

Results: Of the 7,073 total submissions, 5,592 adult blood culture sets were included in the analysis. The Durbin-Watson statistic ranged from 1.92 to 2.37 across models, indicating no significant autocorrelation in residuals. Weekly submission counts declined significantly during the shortage period (p = 0.005) and showed a marginal post-shortage decline (p = 0.052). Single-set submission rates increased significantly during the shortage (p = 0.025) but plateaued afterward. Submission episode counts declined significantly during the shortage period (p = 0.035) and continued to decrease after the shortage (p = 0.034), although no immediate-level change was observed at the shortage onset (p = 0.398). True-positivity rates increased during (p = 0.010) and after (p = 0.028) the shortage, which indicated improved diagnostic yield per test. Contamination rates remained stable throughout the experiment. No significant changes were observed in the distribution of key bloodstream pathogens, such as *Escherichia coli*, *Staphylococcus aureus*, and *Enterococcus* spp., which suggested that the diagnostic quality was maintained.

Conclusions: The bottle shortage prompted fewer submissions, more selective sampling, and higher positivity rates without increased contamination. These findings highlight the value of targeted diagnostic stewardship and the need for adaptable, data-driven protocols to sustain care quality during future supply disruptions.

## Introduction

Blood culture is an essential diagnostic tool for the management of infectious diseases [1]. Blood cultures play a critical role in detecting bloodstream infections (BSIs), identifying causative organisms and drug-resistant pathogens, selecting appropriate antimicrobial therapies, monitoring treatment efficacy, and assessing disease severity [2]. Blood culture positivity rates have been shown to increase with the number of sets collected: 73.2% for one set, 93.9% for two sets, and 96.9% for three sets, indicating that sensitivity improves when moving from the collection of a single set to two or three sets [3]. To maximize diagnostic sensitivity, clinical guidelines such as those from the U.S. Centers for Disease Control and Prevention recommend collecting at least two sets of blood cultures (aerobic and anaerobic bottles) from adult patients [4,5].

Although current guidelines recommend collecting at least two sets to improve diagnostic yield and detect contamination, single-set collections persist in clinical practice, particularly in specific situations. This trade-off between diagnostic accuracy and practicality has come under scrutiny during recent supply shortages, as also emphasized by Fabre et al. in their 2025 publication [6].

In June 2024, a global shortage of BD BACTEC™ blood culture bottles (BD, Franklin Lakes, NJ, US) occurred due to supply chain disruptions at Becton, Dickinson and Company [7]. This shortage significantly affected healthcare institutions across Japan, including the Saitama Medical Center, a tertiary care hospital affiliated with Saitama Medical University, where the supply volume was reduced to approximately 50% of the normal levels. From July 4 to September 21, 2024, we were compelled to implement temporary changes in our blood culture collection practices, including limiting the number of bottles per patient and prioritizing cultures based on the focus of infection, clinical necessity, and severity. Operational decisions had to be made on a case-by-case basis on whether to collect two sets or one set or defer collection. Under these constraints, maintaining diagnostic quality required careful adaptation by clinical staff. In 2024, Itoh et al. reported that their institution adopted a simplified protocol mandating basically single-set collection during the BD BACTEC bottle shortage [8]. Despite effectively streamlining frontline behavior, their approach differed from ours in that it left limited room for clinical discretion. In contrast, our institution prioritized flexibility, allowing physicians to determine the number of sets based on patient-specific factors. Although previous studies have reported the impact of BD blood culture bottle shortages, the feasibility and real-world impact of strategies in healthcare settings remain unclear. Further investigation is warranted, particularly given the considerable variation across institutions in terms of patient demographics, clinical expertise of attending physicians, and routine medical practices [8,9]. In this study, we assessed the impact of a supply shortage in 2024 at our institution by conducting an interrupted time-series (ITS) analysis of weekly blood culture-related indicators. The five target indicators were the total number of submissions, single-set submission rate, number of blood culture submission episodes, true-positivity rate, and contamination rate. In addition, we examined the effect of supply shortages on the detection rates of specific pathogens by comparing the distribution of bacterial species among true-positive cases. This study aimed to provide insights into preparedness planning and support the development of a more resilient diagnostic system by analyzing how the quality of diagnostic practices was influenced by constrained conditions.

## Materials and methods

Study design and setting

This retrospective observational study was conducted at the Saitama Medical Center, a 1,053-bed tertiary care teaching hospital located in Japan. The study period spanned from January 1 to October 31, 2024. Data from all blood culture submissions during this period were extracted from the hospital’s microbiological and electronic health records.

Blood culture bottle supply shortage

Weekly numbers were assigned throughout the study period for comparison. For the analysis, the entire observation period was divided into three phases: pre-shortage (January 1 to July 3, 2024; weeks 1-26), shortage (July 4 to September 21, 2024; weeks 27-38), and post-shortage (September 22 to October 31, 2024; weeks 39-44) periods. The shortage period was defined based on the dates on which the hospital issued internal notifications instructing staff to take measures in response to the limited availability of blood culture bottles and when the supply was restored to normal. The hospital issued the following internal notifications: (1) If antimicrobial therapy did not need to be initiated immediately in patients with fever of unknown origin, blood cultures should be submitted in one set at a time. (2) In patients for whom pneumonia, cellulitis, or urinary tract infection of moderate severity or less was suspected, prioritize sputum or urine cultures, and initiate treatment with narrow-spectrum antibiotics. (3) For follow-up blood cultures, perform one set. (4) If organisms that were contaminants are detected, repeat blood cultures should be avoided, and other potential sources of infection should be identified.

Outcome parameters and definitions

This study evaluated five key outcomes related to blood culture performance: (1) number of blood culture submissions, (2) single-set submission rate, (3) number of blood culture submission episodes, (4) true-positivity rate, and (5) contamination rate. To mitigate biases arising from pediatric blood culture practices, particularly the frequent use of single-set collections in younger patients, the analyses were restricted to individuals aged 15 years and older.

A blood culture set is defined as a pair of culture bottles (aerobic and anaerobic) collected during a single phlebotomy. The single-set submission rate was defined as the proportion of episodes of blood culture collection in which only one set was submitted. If only one set was submitted during an episode, it was classified as a single-set submission.

Single-set submission rate = (Number of episodes with only one set submitted) ÷ (Total number of culture sets submitted)

The total number of submission episodes served as a patient-level metric and was used as the denominator for calculating the true-positivity rate and contamination rate. A submission episode was defined as all blood cultures submitted from the same patient on the same calendar day, regardless of the number of sets submitted.

The true-positivity rate was defined as the proportion of submitted sets that yielded clinically significant bloodstream pathogens. Each submission episode was considered positive if at least one set yielded a recognized pathogen. In patients for whom multiple organisms were recovered from a single submission, the episode was counted as one positive event. Separate submissions from the same patient on different days were considered independent episodes.

True-positivity rate = (Number of episodes with true pathogen growth) ÷ (Total number of submission episodes)

The contamination rate was calculated as the proportion of positive episodes in which only a single set yielded the growth of organisms typically associated with contamination, whereas other simultaneously submitted sets remained negative. To be classified as a contamination episode, the following criteria were required, as described previously with modification [10]: (1) The isolated organisms belonged to a predefined list of likely contaminants: coagulase-negative staphylococci, *Cutibacterium acnes*, *Micrococcus* spp., viridans group streptococci, *Corynebacterium* spp., and *Bacillus* spp. (2) Only one set was positive for the contaminants; at least two sets were submitted from the same patient on the same day. (3) For episodes in which only one set was submitted and the isolate belonged to the pre-defined list of potential contaminants, a case-by-case clinical review was performed by board-certified infectious disease medical doctors. Using comprehensive electronic medical record data (including records documented by the hospital's antimicrobial stewardship team), including clinical presentation, laboratory findings, imaging studies, and therapeutic decisions, each case was adjudicated as either a true BSI or contamination. (4) If a single set was positive for a known pathogen not listed as a potential contaminant (e.g., *Escherichia coli* and *Staphylococcus aureus*), the episode was counted as a true-positive, regardless of the number of sets submitted.

Contamination rate = (Number of suspected contamination episodes) ÷ (Total number of positive episodes)

Comparison of BSI event profiles by bacterial species

We also conducted a comparative analysis of BSI event profiles by bacterial species between the pre-shortage and shortage periods (the post-shortage period was excluded from this analysis because of concerns regarding the appropriateness of combining the pre- and post-shortage periods into a single group for comparison with the shortage period and the relatively short duration of the post-shortage period). The events were restricted to those classified as true positives. For each bacterial species, the number and proportion of true BSI events were calculated separately for the pre-shortage and shortage periods. Fisher’s exact test was used to evaluate whether the distribution of the detected species differed significantly between the two periods.

Statistical analysis

ITS analysis was performed to evaluate the weekly changes in the five outcome indicators between the three periods. Time was treated as a continuous variable, and the model estimated the following effects: an immediate-level change at the onset of the shortage period, a slope change during the shortage period, and a slope change in the post-shortage period. To adjust for potential seasonal variations, the annual periodicity was modeled using sine and cosine terms. Segmented regression was conducted using the Prais-Winsten estimation to account for first-order autocorrelation. Fisher’s exact test was used to compare the detection frequencies of true bloodstream pathogens between the pre-shortage and shortage periods. All statistical analyses were performed using Stata 18.0 BE (StataCorp LLC, College Station, TX, USA), and two-sided p-values < 0.05 were considered statistically significant.

## Results

This study analyzed 7,073 blood culture samples submitted to our microbiology laboratory between January 1 and October 31, 2024. After excluding 1,481 samples obtained from pediatric patients under 15 years of age, 5,592 blood culture submissions from adult patients were retained for the final analysis. After stratifying the submissions by their study period, the number of adult submissions was 3,686 (65.9%) during the pre-shortage phase (January 1 to July 3), 1,192 (21.3%) during the shortage phase (July 4 to September 21), and 714 (12.8%) during the post-shortage phase (September 22 to October 31).

Blood culture submission number

**Figure 1:**
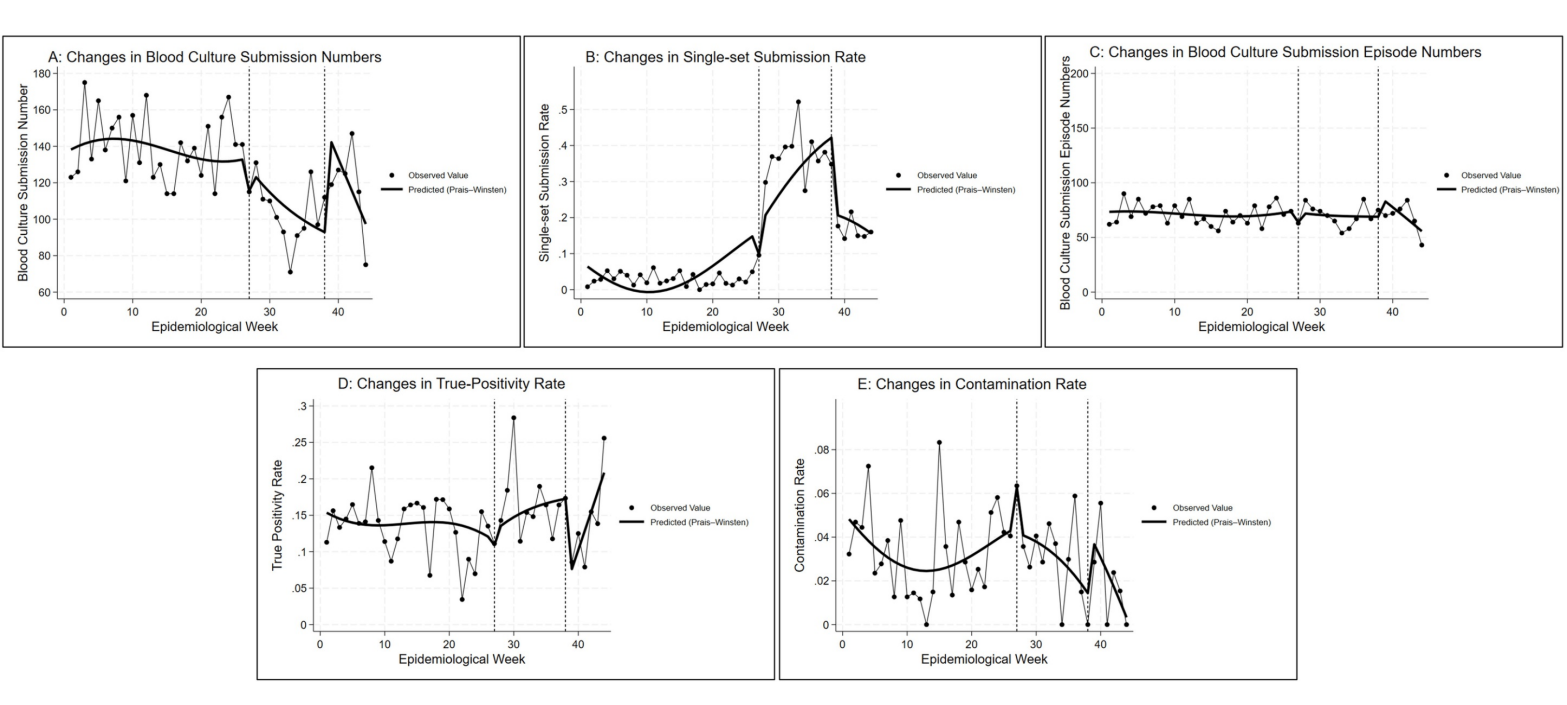
Trends in blood culture metrics before, during, and after the bottle supply shortage (A) Weekly trends in the total number of blood culture submissions, (B) weekly trends in the single-set submission rate, (C) weekly trends in the blood culture submission episode numbers, (D) weekly trends in the true-positivity rate, and (E) weekly trends in the contamination rate from January to October 2024 are shown. Dots represent observed weekly values, and the solid line indicates predicted values based on segmented regression using the Prais-Winsten method. Vertical dashed lines mark the start and end of the blood culture bottle shortage period, corresponding to epidemiological week 27 (beginning July 1, 2024) and week 38 (ending September 21, 2024), respectively.

Table 1 and Figure 1A present the weekly trends in blood culture submission counts. The Durbin-Watson statistic for the model was 1.93, indicating no substantial autocorrelation in the residuals and supporting the validity of the time-series regression approach. Segmented regression using the Prais-Winsten method showed no statistically significant immediate-level change at the onset of the supply shortage (coefficient = −12.1, p = 0.548). However, a significant negative slope was observed during the shortage period (coefficient = −6.0, p = 0.005), indicating a sustained week-to-week decline in submissions. In the post-shortage phase, the decreasing trend appeared to persist, with a marginally significant slope change (coefficient = −13.2, p = 0.052), which may suggest a potential residual effect on submission practices even after the supply issue had been resolved.

**Table 1:** Interrupted time-series regression results for weekly blood culture indicators before, during, and after the bottle supply shortage Results are based on segmented regression using the Prais–Winsten method, which accounts for first-order autocorrelation in weekly time-series data. This table summarizes changes in four blood culture indicators: (1) total number of submissions, (2) single-set submission rate, (3) number of blood culture submission episodes, (4) true-positivity rate, and (5) contamination rate, before, during, and after the blood culture bottle shortage period (epidemiological weeks 27 to 38). Immediate-level change represents the change in the outcome at the onset of the shortage period. Slope change during the shortage indicates the weekly trend change throughout the shortage period. Slope change after the shortage reflects the change in slope following the end of the shortage period. Model p-value reflects the overall statistical significance of each regression model, assessed using the F-test within the Prais-Winsten framework. R^2^ represents the proportion of variance in the outcome variable explained by the model. Statistical significance levels are defined as follows: *p < 0.05, **p < 0.01, and ***p < 0.001.

Outcome variable	Immediate-level change (coefficient (p-value))	Slope change during shortage (coefficient (p-value))	Slope change after shortage (coefficient (p-value))	Model p-value	R^2^
Number of submissions	−12.088 (0.548)	−5.970 (0.005**)	−13.212 (0.052)	0.0003***	0.4827
Single-set submission rate	−0.079 (0.127)	+0.018 (0.025*)	+0.000 (0.998)	0.0000***	0.5845
Number of blood culture submission episodes	−8.803 (0.398)	-2.212 (0.035*)	-7.331 (0.034)	0.2169	0.192
True-positivity rate	−0.019 (0.671)	+0.013 (0.010*)	+0.038 (0.028*)	0.2222	0.19
Contamination rate	+0.021 (0.296)	−0.003 (0.166)	−0.004 (0.531)	0.0366*	0.292

Single-set submission rate

The Durbin-Watson statistic for the single-set submission rate was 2.37, indicating no significant autocorrelation and suggesting the model's residuals were independent. As shown in Table 1 and Figure 1B, the proportion of blood culture episodes with only a single set submitted showed an initial decline at the onset of the shortage; however, this immediate change was not statistically significant (coefficient = −0.079, p = 0.127). In contrast, a significant upward trend was observed during the shortage period (coefficient = +0.018, p = 0.025), reflecting a shift toward more selective sampling practices in response to resource constraints. After the shortage ended, the trend plateaued, with no meaningful change detected in the post-shortage period (coefficient = +0.000, p = 0.998).

Blood culture submission episode number

Table 1 and Figure 1C illustrate the weekly trend in the number of blood culture submission episodes, defined as distinct clinical encounters with at least one blood culture set submitted. The Durbin-Watson statistic was 1.92, indicating no significant autocorrelation in the residuals and confirming the suitability of the segmented time-series model. Segmented regression using the Prais-Winsten method revealed no statistically significant immediate-level change at the start of the shortage period (coefficient = −8.8, p = 0.398). However, a statistically significant decreasing trend was observed during the shortage period (coefficient = −2.21, p = 0.035), suggesting a gradual reduction in the number of blood culture submission episodes week by week. This declining trend appeared to persist into the post-shortage period, with a further significant negative slope (coefficient = −7.33, p = 0.034).

True-positivity rate

Weekly changes in true-positivity rates are presented in Table 1 and Figure 1D. The Durbin-Watson statistic for the model was 1.93, indicating no meaningful autocorrelation, supporting the appropriateness of the regression analysis. There was no significant immediate-level change following the onset of the shortage (coefficient = −0.019, p = 0.671). However, a significant upward trend was observed during the shortage period (coefficient = +0.013, p = 0.010), suggesting an improvement in diagnostic yield per test. This positive trend appeared to persist into the post-shortage period (coefficient = +0.038, p = 0.028), potentially reflecting more judicious patient selection for blood cultures by clinicians.

Contamination rate

The Durbin-Watson statistic for the contamination rate was 2.05, suggesting no substantial autocorrelation in the residuals and supporting the validity of the regression model. As summarized in Table 1 and illustrated in Figure 1E, contamination rates remained stable, with no statistically significant changes observed throughout the study period. The immediate-level change at the onset of the shortage was not significant (coefficient = +0.021, p = 0.296), nor were the trends during (coefficient = −0.003, p = 0.166) or after the shortage (coefficient = −0.004, p = 0.531). These findings indicate that specimen quality, as measured by contamination rates, was likely preserved despite the supply disruption.

Comparison of BSI event profiles by bacterial species

A comparison of the distribution of true BSI pathogens before and during the shortage period is summarized in Table 2. *E. coli* and *S. aureus* were the most frequently isolated organisms, and no statistically significant differences in detection frequencies were identified between periods. Similarly, other clinically relevant pathogens, such as *Klebsiella* spp., *Enterococcus* spp., *Enterobacter cloacae* complex, *Pseudomonas aeruginosa*, and *Candida* spp., did not exhibit statistically significant changes between study periods. To address the potential overrepresentation of persistent bacteremia, a sensitivity analysis was conducted by collapsing multiple positive detections of the same organism from the same patient within a one-week interval into a single event (data not shown). The findings of this reanalysis are consistent with those of the primary analysis, reinforcing the robustness of the observed distribution patterns.

**Table 2:** Changes in detection rates of bloodstream pathogens during the blood culture bottle supply shortage GPC: Gram-positive cocci; GPR: Gram-positive rods; GNC: Gram-negative cocci; GNR: Gram-negative rods The table summarizes the number and percentage of true-positive blood culture episodes for each organism group before and during the shortage of blood culture bottles. Each episode was defined as a unique patient day with a confirmed bloodstream infection. If multiple species were identified in a single submission, a single episode was considered. Only patients aged ≥15 years were included, and duplicate episodes were removed based on patient ID and date. Comparisons between the pre-shortage and shortage periods were performed using Fisher’s exact test. p-values less than 0.05 were considered statistically significant.

Strain	Pre-shortage count (n = 350)	Shortage period count (n = 154)	p-value
Gram-positive bacteria			
*Bacillus* spp.	2	3	0.169
*Clostridium *spp.	11	2	0.361
*Corynebacterium *spp.	4	2	1
*Cutibacterium *spp.	1	0	-
*Enterococcus *spp.	35	12	0.508
Staphylococcus aureus	54	21	0.684
Other *Staphylococcus *spp.	34	18	0.526
*Streptococcus *spp.	26	10	0.851
Other GPC	3	3	0.376
Other GPR	5	3	0.705
Gram-negative bacteria			
*Acinetobacter* spp.	2	1	1
*Bacteroides *spp.	9	1	0.296
*Citrobacter *spp.	5	1	0.672
*Enterobacter cloacae* complex	14	11	0.179
Escherichia coli	57	23	0.792
Haemophilus influenzae	1	0	-
*Klebsiella *spp.	38	22	0.297
*Proteus *spp.	4	1	1
Pseudomonas aeruginosa	14	7	0.810
Salmonella enterica	1	2	0.223
Serratia marcescens	3	0	-
Other GNC	1	1	0.518
Other GNR	18	7	1
Fungi			
*Candida *spp.	7	3	1
Other fungi	1	0	-

## Discussion

This study investigated the institutional impact of a temporary supply shortage of BD BACTEC™ blood culture bottles on diagnostic practices at the Saitama Medical Center, a university-affiliated tertiary care hospital in Japan. In response to the unexpected disruption in bottle availability in mid-2024, institutional practices were promptly revised to optimize resource utilization without compromising diagnostic quality. A more selective, indication-driven approach to blood culture collection was implemented, whereby single-set collections were recommended when deemed clinically sufficient, based on the suspected focus of infection, patient acuity, and overall diagnostic rationale.

Our findings revealed that the number of blood culture submissions did not decline immediately after the onset of the shortage (Table 1 and Figure 1A). However, the weekly submission volume decreased significantly over the course of the shortage and appeared to continue to decline even after supply restoration. This pattern suggests a delayed but sustained behavioral adaptation among clinicians, which likely reflected an increased awareness of resource constraints and compliance with internal guidance. These findings support the concept that institutional messaging can substantially influence clinical decision-making, especially when implemented through coordinated communication strategies and reinforced by accountability mechanisms across the healthcare system. Similar trends were documented by Ryder et al., who observed prompt and measurable changes in blood culture submission practices during bottle shortage after the implementation of targeted diagnostic stewardship interventions [9].

A notable shift was observed in the single-set submission rates (Table 1 and Figure 1B). Following an insignificant initial decline, the rate of single-set collections increased significantly during the shortage period. This change reflected the successful implementation of the revised protocols and suggests that under conditions of resource scarcity, clinicians adapted by selectively applying single-set sampling when clinically appropriate, balancing efficiency with diagnostic adequacy. Similar findings have been reported in an intensive care unit (ICU)-based intervention study, in which adoption of a single-venipuncture strategy reduced contamination by approximately 60% without compromising BSI detection [11]. Interestingly, the increase appeared not to be sustained in the post-shortage period. This temporal pattern underscores the responsiveness of frontline healthcare workers to institutional policy and highlights the importance of clear and temporally bound guidance in driving behavioral change.

In contrast to the notable decline in the total number of culture sets submitted, the number of blood culture submission episodes, defined as distinct clinical encounters, remained relatively stable throughout the study period (Table 1 and Figure 1C). Although segmented regression revealed a statistically significant downward trend during and after the shortage, the overall trajectory appeared to visually plateau when compared to the marked changes observed in other metrics. This pattern can be interpreted as a compensatory effect: the decrease in multiset submissions was partially offset by a corresponding increase in single-set submissions. Consequently, while clinicians responded to the shortage by reducing bottle usage per episode, the overall frequency of diagnostic sampling at the patient level remained largely unchanged. This observation suggests that diagnostic vigilance was preserved even under constrained conditions and that clinicians may have prioritized efficiency without compromising the threshold for initiating blood cultures. From a stewardship perspective, these findings emphasize that episode-level submission metrics offer a complementary lens to understand clinical behavior, especially in settings where both overuse and underuse must be carefully balanced.

Importantly, the true-positivity rate increased significantly during the shortage period and appeared to continue to increase during the post-shortage period (Table 1 and Figure 1D). This observation suggests that clinicians may have exercised greater discretion in selecting patients for blood culture during this period, which may have improved diagnostic efficiency. Despite the overall reduction in the number of submitted cultures, the yield per test improved, thus indicating the need for more targeted sampling. This observation is consistent with the hypothesis that diagnostic stewardship, whether explicitly framed or indirectly induced by resource limitations, improves the clinical utility of diagnostic testing.

However, caution is warranted when interpreting positivity rate trends, as increased rates may also reflect under-testing or missed cases of low-grade bacteremia. Previous studies have shown that diagnostic stewardship interventions can improve blood culture positivity rates while reducing unnecessary testing. For example, Fabre et al. reported an increase in positivity from 8% to 11% following a targeted intervention in ICUs [12], and Wang et al. reported that positivity was maintained despite reduced testing volumes [13]. These findings support our observation that resource constraints enhance diagnostic efficiency through selective test utilization.

The contamination rate remained stable across all three periods, with no statistically significant level or trend changes (Table 1 and Figure 1E). This stability was achieved despite an increase in single-set collections, which are often associated with a higher contamination risk owing to the absence of internal validation through paired sets. The absence of increased contamination suggests that the staff adhered to proper collection techniques possibly supported by enhanced awareness, stricter adherence to aseptic protocols, or additional training prompted by the supply constraints. Seasonal adjustments were included in our statistical model to account for cyclical variations, and the seasonal term was significant only in the contamination model. The stability of the contamination rate was in line with previous findings, whereby education and procedural bundles significantly reduced contamination events [14,15]. However, the methodological limitations of single-set protocols, including the inability to identify contaminants without a paired negative comparator, echo concerns raised by previous meta-analyses [16].

Our findings in this study contrast with those reported by Itoh et al., who implemented a hospital-wide policy mandating single-set blood culture collection during the 2024 shortage [8]. Although their one-set submission approach effectively minimized bottle usage and maintained submission volume, it may have reduced sensitivity and precluded accurate contamination assessment, as reflected in their observed decline in positivity rate and zero contamination rate. In our setting, however, although institutional announcements were made to promote adherence to recommended sampling criteria, we allowed clinicians to retain discretion regarding the number of sets per episode, recognizing the importance of individualized clinical judgment. This flexibility may have helped preserve diagnostic accuracy while still achieving reductions in bottle consumption through selective adaptation. These differences highlight the importance of balancing system-level directives with frontline clinical judgment, especially during supply-constrained periods.

We also analyzed changes in the distribution of bloodstream infectious pathogens (Table 2). No statistically significant shifts were observed in the detection frequencies of major organisms such as *E. coli* and *S. aureus*, suggesting that the prioritization of blood culture collection in patients with a higher likelihood of bacteremia was effective in maintaining coverage of infections due to clinically important pathogens. Sensitivity analyses that excluded recurrent detections from persistent bacteremia yielded results that were consistent with those of the main analysis, thus indicating the robustness of the findings and minimal distortion due to repeat events (data not shown). A similar finding was reported by Sinto et al., who observed no substantial changes in the distribution of major bloodstream pathogens such as *Klebsiella pneumoniae*, *E. coli*, and *S. aureus* before and during the COVID-19 pandemic at a national referral hospital in Indonesia [17].

The overall model fit varied across the outcomes that were evaluated in this study. The regression model for the single-set submission rate showed strong explanatory power (R² = 0.5845, p < 0.001), thus suggesting that time-series trends and seasonal adjustments accounted for a substantial proportion of the variance. In contrast, the model for the contamination rate revealed a relatively low explanatory strength (R² = 0.366, p = 0.0292), which potentially reflects the stochastic nature of contamination events and the limitations of ascertainment during single-set collections. The true-positivity rate model yielded a modest R² of 0.2222 (p = 0.190), which may indicate that although a significant temporal trend was present, much of the variability remained unexplained, possibly due to clinical factors not captured in this dataset, such as patient severity. These differences in model fit highlight the multifactorial nature of diagnostic outcomes in real-world settings and suggest that while submission behaviors may be influenced by institutional policies and time-dependent interventions, microbiological outcomes such as positivity and contamination rates may reflect a broader range of clinical and operational variables not fully captured in routine datasets. While temporal patterns were evident, particularly for submission behaviors, microbiological outcomes such as positivity and contamination rates may be influenced by a broader range of clinical, procedural, and institutional variables not included in the current analysis [10].

This study had several limitations that should be acknowledged when interpreting our results. This single-center, retrospective observational study was conducted at a university hospital, and the findings, therefore, may not be generalizable to other healthcare settings. Institutional characteristics, including academic affiliation, resource availability, and a culture of quality improvement, may influence response strategies and outcomes. Moreover, clinical outcomes such as delayed diagnoses, treatment failures, and patient mortality were not assessed. The study period spanned 10 months and did not capture a full seasonal cycle, although seasonality was statistically adjusted. The duration of the post-shortage observation period was relatively short, which limits our ability to assess the long-term sustainability of the observed changes. To address these limitations, a follow-up extending the observation period and incorporating detailed clinical parameters is currently under consideration.

## Conclusions

In conclusion, our findings indicate that moderate but measurable changes in blood culture practices occurred during the 2024 blood culture bottle shortage at our institution. The shortage prompted measurable changes in approach for blood culture requests, which included a temporary decline in submission volume, a significant increase in single-set sampling, and a statistically significant increase in true-positivity rates. Notably, these changes occurred without a corresponding increase in contamination rates. The detection spectrum of major pathogens remained stable, suggesting that diagnostic quality had not been markedly compromised. However, reliance on single-set collections may limit contamination monitoring and reduce diagnostic sensitivity for certain pathogens. As healthcare systems prepare for future resource disruptions, our experience underscores the potential utility, but also the limitations, of adaptive protocols implemented under emergency conditions. Given that healthcare institutions differ widely in terms of size, patient population, and provider characteristics, it is crucial that each facility reflects on its own experience during past disruptions. Proactively simulating tailored response strategies to anticipated medical crises, based on each institution's specific context, will be essential for strengthening resilience and preparedness.
